# ELECTRUM: an electron configuration-based universal metal fingerprint for transition metal compounds

**DOI:** 10.1039/d5dd00145e

**Published:** 2025-10-20

**Authors:** Markus Orsi, Angelo Frei

**Affiliations:** a Department of Chemistry, Biochemistry & Pharmaceutical Sciences, University of Bern Freiestrasse 3 3012 Bern Switzerland markus.orsi@unibe.ch; b Department of Chemistry, University of York York YO10 5DD UK angelo.frei@york.ac.uk

## Abstract

Machine learning has experienced a drastic rise in interest and applications in all fields of chemistry, enabling researchers to leverage large chemical datasets to gain novel insights. The success of machine learning-driven projects in chemistry hinges on three key factors: access to robust and comprehensive datasets, a well-defined objective, and effective molecular representations that convert chemical structures into machine-readable formats. Transition metal complexes have lagged behind their organic counterparts on all three of these avenues. The large diversity of structures, coordination numbers and modes have made its translation to a machine-readable format an ongoing challenge. Here we introduce ELECTRUM, an electron configuration-based universal metal fingerprint for transition metal compounds. Its lightweight implementation enables the straightforward conversion of any transition metal complex into a simple fingerprint. Utilising a novel dataset generated from the Cambridge Structural Database (CSD), we demonstrate that ELECTRUM effectively captures the structural diversity of transition metal complexes. By plotting nearest-neighbor relationships in ELECTRUM space, we reveal meaningful clustering in two-dimensional representations. Furthermore, we use the ELECTRUM encoding to train machine learning models on the prediction of metal complex coordination numbers from ligand structures and metal identity alone. We show that on a subset of this data, we can train models to predict the oxidation state of metal complexes. These case studies showcase the potential of ELECTRUM as an easy-to-implement fingerprint for metal complexes. We rely on the community to further test, validate, and improve it.

## Abstract 2

ELECTRUM is a lightweight, electron-configuration-based fingerprint for transition-metal complexes. Validated on CSD-derived datasets, it captures structural diversity and enables ML to predict coordination numbers and oxidation states.

## Graphical abstract

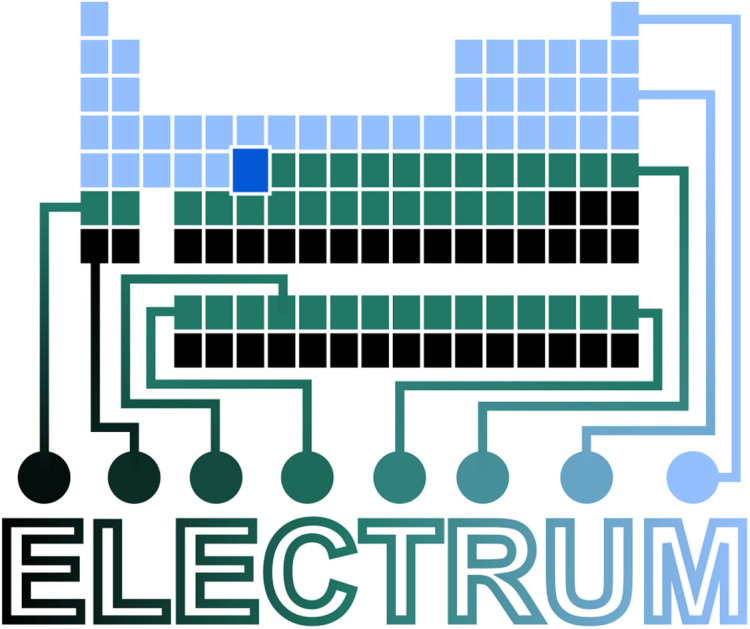

## Introduction

The emergence of automated high-throughput synthesis and testing in the chemical sciences has led to data-driven approaches gaining significant traction, taking advantage of the increased availability of data. Consequently, a wide array of machine learning (ML) techniques has been developed to extract insights from this data and tackle various bottlenecks in materials and drug discovery processes. Many of these techniques aim to identify potential new candidate compounds by predicting critical physical, chemical, and/or biological properties.^1–18^ However, for ML approaches in chemistry to be effective, a few criteria need to be fulfilled: (1) high-quality data in the relevant domain must be available, (2) the objective function must be clearly defined, and (3) molecules must be translated to a machine-readable format that contains all necessary molecular information for the model to learn the objective function. Each of these criteria presents its own set of challenges. Although high-quality datasets of organometallic compounds are becoming more accessible, they remain relatively rare and are often smaller than those available for medicinal chemistry. Moreover, defining clear objective functions is challenging in areas like materials and drug development where multiple properties need to be optimized simultaneously.^19,20^ Finally, the translation of molecules to machine-readable formats has been addressed in many different ways, including pharmacophore descriptors (CATS,^21^ MXFP^22,23^), substructure and atom-pair descriptors (ECFP,^24^ Atom-Pair,^25^ MAP4C^26^) as well as graph-based approaches.^27–31^ However, this progress has mostly been restricted to organic molecules, which are mainly carbon-based and generally adhere to covalency rules. In contrast, transition metal compounds have largely been excluded from these methods due to their diverse binding modes, oxidation states, and geometries, which current approaches cannot comprehensively encode. Given the widespread application of transition metal complexes in catalysis, luminophores, materials, medicine, and more, a suitable molecular representation that encompasses all these possible compounds would be highly desirable.

In recent years, several notable efforts have been made in this direction, primarily within the area of catalyst development.^32–34^ Although in some cases a single scalar descriptor may suffice to correlate with the desired outcome, higher-dimensional descriptors that incorporate experimentally determinable or computationally derived properties of metal complexes often yield better predictive performance.^35^ For instance, MCDL-25 descriptors used in conjunction with artificial neural networks (ANNs) have been used to predict quantum-mechanically derived properties of metal complexes.^36^ Since then, the field has seen a broad expansion of various encodings and machine learning models designed to accelerate the discovery of inorganic materials and catalysts.^37–45^

All these approaches represent promising strides forward; however, their implementation at scale and by non-experts is not trivial. Graph-based encodings, although detailed, may suffer from scalability and complexity issues, while string-based representations often lack the ability to fully capture the multifaceted chemistry of metal complexes. Other representations depend heavily on descriptors derived either experimentally or through computationally intensive methods. Generally, however, these representations all require a structure of the compound available as a .mol or .xyz file to generate the respective descriptors. Molecular fingerprints, in contrast, provide an efficient and easily interpretable way to encode essential structural features, offering excellent scalability and versatility. They are widely employed in cheminformatics tasks such as virtual screening and compound database searches due to their simplicity and efficiency.

Based on this quandary and our own needs to have a suitable descriptor for the prediction of the biological properties of metal complexes^3^ we have developed our own approach. Herein we present the Electron Configuration-based Universal Metal Fingerprint for transition metal compounds (ELECTRUM). ELECTRUM takes into account the nature of any ligands coordinated to the metal complex as well as the electronic properties of the specific metal to generate a 598-bit fingerprint. Importantly, ELECTRUM fingerprints can be generated from simple concatenated strings of the involved metal center and ligand SMILES. We show that this fingerprint can be applied to a series of classification tasks and provide a new CCSD dataset for the prediction of transition metal complex coordination numbers. Our hope is that ELECTRUM will be taken up, applied and improved by the community going forward to provide researchers with an easy to implement fingerprint for transition metal-based ML projects.

## Results and discussion

### Fingerprint design

**Fig. 1:**
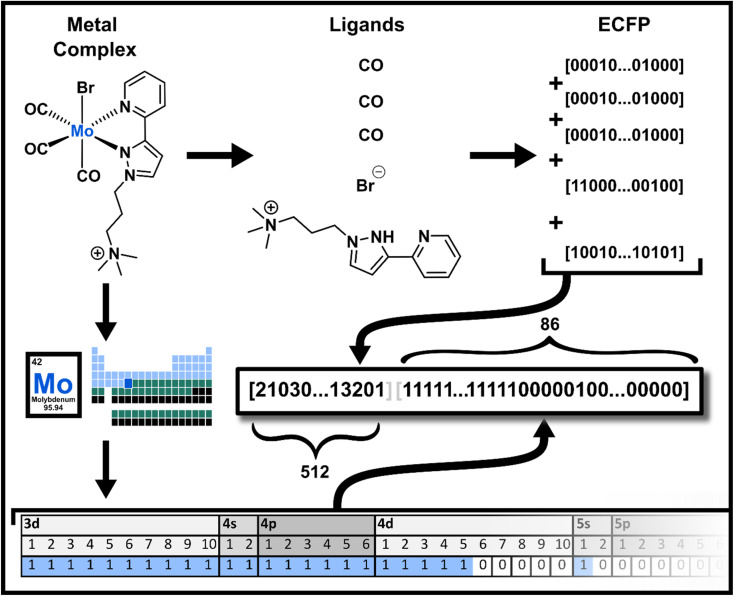
Concept of ELECTRUM. The metal complex is divided into its ligands and the central metal. Each ligand is encoded using an ECFP-like hashing and folding method, resulting in fingerprints that are combined bitwise to produce a 512-bit ligand fingerprint. This fingerprint is then augmented with an 86-bit representation of the electron configuration of the coordinating metal, creating the final 598-bit ELECTRUM fingerprint.

The effectiveness of molecular representations for metal complexes can be significantly improved by explicitly incorporating ligand-specific information along with electronic properties of the central metal.^36^ Previously, we demonstrated that bitwise summation of ECFP-like fingerprints for ligands (Ligands fingerprint) within a metal complex effectively captures ligand combinations; however, this approach was limited because it only allowed comparisons among complexes containing the same central metal.^3^ To overcome this limitation, we now extend this method by appending explicit information about the electron configuration of the metal center (Fig. 1). Consequently, generating ELECTRUM fingerprints only requires the SMILES strings of individual ligands along with the identity of the coordinating metal.

ELECTRUM fingerprints are calculated starting from the metal and ligands, which are represented in SMILES format, concatenated and separated by dots (“SMILES1.SMILES2.SMILES3”). For each ligand, circular substructures are extracted up to a radius of 2 bonds from each atom, capturing the local chemical environments. The substructures are then hashed to generate integer identifiers, which are folded using a modulo operation to obtain a fixed-size vector representing the ligand. In this study, we have opted for a bit size of 512, which is lower than the typical bit sizes used for drug discovery. We chose this size because ligand structures are typically small, and a lower bit size is sufficient to encode metal complexes. Additionally, we hypothesize that a lower feature number may benefit machine learning models by reducing dimensionality. Once the folded fingerprints for all ligands are obtained, they are combined through bitwise summation. This procedure retains information about both the complex and the individual ligands, including instances where the same ligand appears multiple times, as the bitwise summation reflects the cumulative presence of substructures. Hence, there is no information loss linked to repeated ligand appearance. Additionally, compared to concatenation methods, bitwise summation produces a permutation-invariant descriptor, better reflecting the structural characteristics of metal complexes. Finally, we append an 86-bit binary representation of the coordinating metal's electron configuration to the ligand fingerprint, resulting in the final 598-bit ELECTRUM fingerprint.

One of ELECTRUM's key advantages is its low computational cost relative to geometry-based descriptors and quantum-derived features. Fingerprint generation scales linearly with the number of atoms in the ligand set, O(*N*), as it involves circular substructure enumeration and hashing, plus a constant-time 86-bit electron configuration encoding for the metal center. By contrast, many geometry-dependent methods require full coordinate sets and, in some cases, geometry optimization, which can scale superlinearly with system size. Even 3D descriptors derived directly from crystallographic coordinates typically involve distance matrix computations with O(*N*^2^) scaling. This difference in complexity makes ELECTRUM particularly interesting for larger metal complexes or tasks where high throughput is a priority. In more practical terms, generation of 217 517 fingerprints (ligand radius = 2, ligand fingerprint size = 512, full 86-bit electron-configuration appended) on a single Apple M1 Pro chip (10-core CPU, 16 GB RAM.) required 265 s (≈4.4 min), corresponding to ≈0.0012 s (1.2 ms) per complex. Timings correspond to a single process run and include SMILES parsing, substructure enumeration, hashing/folding and metal vector concatenation. Although not directly comparable, many metal complexes descriptors require the generation of high-quality 3D conformers. State-of-the-art tools such as Architector^46^ can require ≈1 h per complex on a single CPU core, highlighting that ELECTRUM can achieve speedups of 10^3^–10^6^ per complex compared to conventional 3D/QM pipelines, making it better suited for early screening and big data-driven discovery.

Next, we aimed to validate the usefulness of ELECTRUM through a series of test cases. In each of these test cases, we benchmarked ELECTRUM against two alternatives, (i) the ligands fingerprint (Ligands) without metal encoding as a negative control or (ii) the ligands fingerprint + a single scalar metal identifier (Atomic). Additionally, for each fingerprint we tested different ligand fingerprint bit-sizes of 256, 512 and 1024 bits.

### Model selection

We chose a Multilayer Perceptron (MLP) neural network as our model for this study.^47^ Given the demonstrated effectiveness of artificial neural networks (ANNs) in predicting quantum-mechanical properties^36^ and our successful use of MLPs in prior research,^3^ we chose this architecture to evaluate the performance of the ELECTRUM fingerprint. Additionally, MLPs are particularly well-suited for processing high-dimensional input data, such as the 598-bit ELECTRUM fingerprints. The model was implemented in Python using the scikit-learn library. We configured the MLP with 5 hidden layers, with the number of neurons per layer decreasing from 512 to 256, 128, 64, and finally 32. For each task, we evaluated the model using 5-fold cross-validation, comparing performance on both true and randomly scrambled labels. Using scrambled labels as a negative control helps verify that the model's performance is not due to chance or overfitting, ensuring it learns meaningful patterns related to the target variable. For classification tasks, we report the area under the receiver operating characteristic curve (AUROC), area under the precision–recall curve (AUPRC), accuracy, precision, recall, and F1 score, calculated as macro-averaged metrics.

#### Test case 1: coordination number prediction

As a first test case, we extracted a novel dataset from the CSD to evaluate the ability of ELECTRUM to predict the coordination number of transition metal complexes. For this, we filtered the CSD for mononuclear metal complexes containing any of the following elements: Bi, Cd, Ce, Cr, Co, Cu, Dy, Er, Eu, Gd, Ga, Ge, Au, Hf, Ho, In, Ir, Fe, La, Pb, Lu, Mn, Hg, Mo, Nd, Ni, Nb, Os, Pa, Pt, Pr, Re, Rh, Ru, Sm, Sc, Ta, Tc, Tb, Tl, Tm, Sn, Ti, W, V, Y, Zn, Zr. Counterions were removed, and utilizing *RDKit*'s MolFromSmiles function without sanitization, we generated workable molecule objects from the deposited SMILES. From these, we created adjacency matrices using the *networx* library. We then removed the metal ion from the adjacency matrix and converted the resulting network back to a molecule object and then to SMILES format. This process resulted in concatenated SMILES strings of all the ligands in each transition metal complex. During this process, we also extracted and stored the number and types of bonds between the metal and the ligands. For the dataset, we chose to keep only the coordination numbers for which at least 1000 examples were present to ensure that each class had enough data for training the model effectively. Through this, we obtained a dataset of 217 518 transition metal complexes with their respective coordination number.

The dataset contained 35 metals and 11 distinct coordination numbers, providing sufficient chemical diversity despite large data imbalances. The metal counts are dominated by Cu, Ru, Fe, Ni, and Pd (88 657 entries, 40.8%, Fig. S1a), and the coordination numbers by 6, 4, and 5 (164 496 entries, 75.6%, Fig. S1b). These patterns likely reflect constraints in crystallography and synthetic chemistry: certain metals and geometries are both more synthetically accessible and more amenable to single-crystal diffraction, leading to their over-representation in the CSD. For instance, Pt(ii) and Pd(ii) complexes frequently adopt square-planar (CN 4) geometries, while Ru(ii), Re(i) and Ir(iii) complexes often occur in octahedral coordination. At the same time, the dataset includes a wide range of less common metal-coordination number combinations (Fig. S1c), ensuring that ELECTRUM is evaluated across a structurally varied space that also mirrors real-world data availability rather than an artificially uniform distribution. The dataset and code is available on our GitHub repository.^48^

**Fig. 2:**
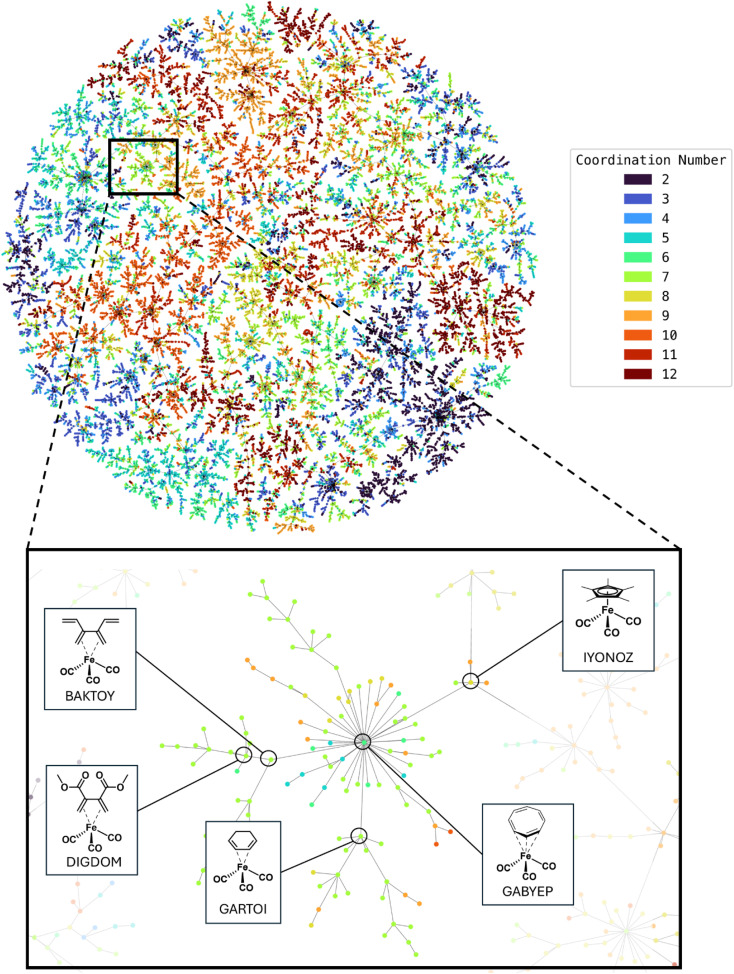
(Top) TMAP of 22 000 compound subset of coordination number dataset, showing clustering of compounds based on ELECTRUM fingerprint similarities and coordination numbers. (Bottom) Example dendritic structure with selected metal complex-structures shown. Interactive version available on GitHub.^48^

We encoded all complexes in the dataset using ELECTRUM and computed nearest neighbor relationships based on the 598-dimensional fingerprints. To visualize these relationships in two dimensions, we employed TMAP visualization (Fig. 2). TMAP is a visualization method that builds a minimum spanning tree from the *k*-nearest neighbor graph of input vectors, which can be based on a user-defined distance metric. It acts as a dimensionality reduction technique that preserves both local and global relationships of high-dimensional data. This allows the structure of the original space to be reflected in two-dimensional layouts, where spatial proximity implies similarity. In this study, we computed the *k*-nearest neighbor graph of the ELECTRUM fingerprints using the Manhattan distance, which guided how compounds were arranged in the final TMAP plot. The TMAP visualization revealed clear clustering of compounds according to their coordination numbers, indicating that the ELECTRUM fingerprint effectively captures structural similarities relevant to coordination. In earlier studies, greater distances in low-dimensional visualizations corresponded to difficulties in predicting specific properties.^42^ Consequently, a clear, structured clustering in such visualizations suggests that the high-dimensional fingerprint space suitably captures relevant structural information. The clear clustering observed in our TMAP visualization indicates that the MLP model is likely capable of effectively learning to predict coordination numbers from structural data encoded by ELECTRUM fingerprints, as complexes with the same coordination number cluster closely together. The closer inspection of the coordination number TMAP reveals that complexes are clustered generally by ligand type first and by metal type second, which makes sense when one considers that the ligand-part of the fingerprint is clearly longer. An example is highlighted in Fig. 2, with the core structure an iron cyclooctatetraene tricarbonyl complex (GABYEP). The highlighted examples show that the surrounding structures feature quite similar iron tricarbonyl compounds with different types of ligands that generally bond *via* π-orbitals. The reader is encouraged to explore the interactive version of the TMAP which is accessible on our GitHub repository.^48^

Although the coordination number can be directly extracted from structural data, we use its prediction as a proxy to assess whether ELECTRUM captures chemically meaningful features of the coordination environment which are not explicitly encoded in the fingerprint. In the prediction task, ELECTRUM outperforms both baseline fingerprints (Ligands, Atomic) across all evaluated metrics for coordination number prediction (Table 1). The ROC AUC improves from 79.7% with the Ligands fingerprint baseline to 85.7%; the PRC AUC rises from 44.3% to 58.9%; the precision increases from 64.7% (Ligands) and 67.3% (Atomic) to 75.9% and recall improves from 62.3% and 64.9% to 73.4%. For all tested metrics, the performance gain from the Ligand fingerprint to the Atomic fingerprint is modest, but the improvement achieved by ELECTRUM is much larger, indicating that inclusion of the full binary electron configuration of the metal center provides significant additional predictive signal beyond ligand identity alone. Overall, the good performance metrics achieved in the predictive task, combined with the well-structured CN-based clusters in the TMAP visualization, indicate that ELECTRUM encodes relevant information about metal–ligand connectivity even without explicitly representing the coordination bonds.

**Table 1:** Performance metrics for coordination number classification using the MLP model and ELECTRUM fingerprint (598 bits) compared to the ligands fingerprint without metal encoding (Ligands) as a negative control and the ligands fingerprint + a single scalar metal identifier (Atomic). Metrics for shorter and longer fingerprints can be found in the SI

Fingerprint	ROC AUC	PRC AUC	Precision	Recall	F1
Ligands	79.7 ± 0.45	44.3 ± 0.10	64.7 ± 1.33	62.3 ± 0.90	63.3 ± 0.08
Atomic	81.1 ± 0.34	47.5 ± 0.36	67.3 ± 0.68	64.9 ± 0.66	65.9 ± 0.32
ELECTRUM	**85.7** ± **0.63**	**58.9** ± **0.55**	**75.9** ± **0.48**	**73.4** ± **1.26**	**74.5** ± **0.55**

#### Test case 2: oxidation state prediction

Utilising a subset of the Cambridge Structural Database (CSD) provided by Jensen and co-workers^49^ we generated a dataset of 38 778 monometal complexes together with the oxidation state of the metal.^50^ Briefly, we took the intersection of the set provided by Jensen and our own to ensure that all included compounds would correspond to the criteria set out in the first test-case and have a workable SMILES string for processing. Predicting the oxidation state of transition metal complexes is a fundamental task in inorganic chemistry and is essential for understanding reactivity and other chemical properties. The dataset comprised 29 metals (Mn, Ag, Cu, Au, Re, Rh, Ir, Co, Tc, Y, Nb, Sc, V, Ta, Ru, Cr, Pd, Pt, Mo, Ni, Fe, Ti, Os, Zn, Hg, Cd, Zr, W, Hf) spanning seven oxidation states (0, +1, +2, +3, +4, +5, +6), providing broad chemical coverage despite data imbalances. The metal distribution is concentrated on a few elements, with Pd, Ni, Pt, Zn, and Ru accounting for 18 803 complexes (48.0%, Fig. S2a). Similarly, oxidation states +2, +1, and +4 constitute the majority of the dataset, together covering 30 542 entries (78.0%, Fig. S2b). These patterns reflect well-known chemical preferences, for example, Pd(ii), Pt(ii), and Ni(ii) complexes are ubiquitous in organometallic chemistry. The cross-tabulation of metals and oxidation states (Fig. S2c) reveals strong associations, with certain metals occurring almost exclusively in a single oxidation state.

**Fig. 3:**
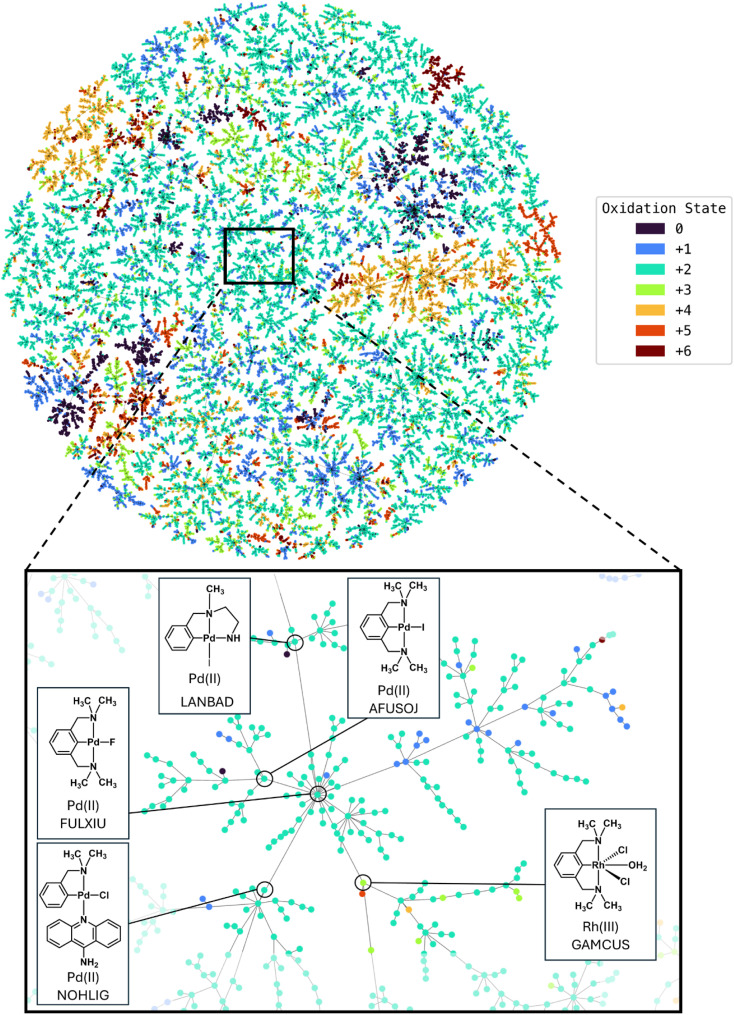
(Top) TMAP of oxidation state dataset (39 166 compounds), showing clustering of complexes based on ELECTRUM fingerprint similarities and oxidation states. Interactive version available on GitHub. (Bottom) Example dendritic structure with selected metal complex-structures shown. Interactive version available on GitHub.^48^

We encoded all complexes in the dataset using ELECTRUM and computed nearest neighbor relationships based on the 598-dimensional fingerprints. To visualize these relationships in two dimensions, we employed TMAP visualization (Fig. 3). Similar to the first test case, the TMAP revealed clear clustering of compounds according to their oxidation states. This indicates that ELECTRUM effectively captures structural features relevant to oxidation state, as compounds with the same oxidation state are positioned close to each other in ELECTRUM space. An example is highlighted in Fig. 3. The core structure is a square planar palladium(ii) pincer complex. Highlighted examples around the core show that compounds that are proximal in this space show both similar ligand structures as well generally the same or similar oxidation states. The reader is encouraged to explore the interactive version of the TMAP which is accessible on our GitHub repository.^48^

Overall, the oxidation state classification task demonstrated very strong performance for ELECTRUM, with the ROC AUC reaching 95.1%, PRC AUC 85.2%, and precision, recall, and F1 scores all exceeding 91% for true labels (Table 2). These results represent a marked improvement over both Ligand and Atomic fingerprints, indicating that explicit electron configuration encoding provides substantial predictive value. The high recall (91.2%) shows that the correct oxidation state is recovered for the vast majority of cases, while the equally high precision (92.5%) confirms that false positives are rare. The balanced F1 score (91.8%) highlights robust classification performance across all oxidation state classes, despite class imbalance in the dataset.

**Table 2:** Performance metrics for coordination number classification using the MLP model and ELECTRUM fingerprint (598 bits) compared to the ligands fingerprint without metal encoding (Ligands) as a negative control and the ligands fingerprint + a single scalar metal identifier (Atomic). Metrics for shorter and longer fingerprints can be found in the SI

Fingerprint	ROC AUC	PRC AUC	Precision	Recall	F1
Ligands	79.8 ± 0.2	48.2 ± 0.8	66.8 ± 0.9	64.9 ± 0.3	65.7 ± 0.6
Atomic	82.2 ± 0.5	54.0 ± 0.4	72.4 ± 0.2	69.2 ± 0.7	70.6 ± 0.4
ELECTRUM	**95.1** ± **0.3**	**85.2** ± **0.6**	**92.5** ± **1.2**	**91.2** ± **0.5**	**91.8** ± **0.4**

For context, while direct numerical comparison is not possible due to different datasets and evaluation setups, our results are in the same general performance range as other state-of-the-art approaches. For example, Jablonka *et al.*^51^ report oxidation state classification precision and recall above 95% using models that incorporate also the local coordination geometry. Similarly, the cell2mol framework of Vela *et al.*^52^ achieves comparable overall classification performance, although their reported accuracy metric is not directly transferable to our reported metrics. In contrast to these methods, ELECTRUM requires only 2D ligand SMILES strings and a simple binary encoding of the metal's electron configuration, and we performed no hyperparameter tuning in these experiments. That ELECTRUM can approach the performance of geometry-aware, fully optimised models under these constraints suggests that it is able to capture chemically relevant information about the coordination environment from minimal input. Ultimately, the focus here is not on developing a new state-of-the-art oxidation state predictor, but on using these predictive tasks as proxies to demonstrate that ELECTRUM encodes chemically meaningful features that can support a broad range of downstream applications.

#### Test case 3: prediction of quantum mechanical properties

As a final test case we downloaded the tmQMg dataset^53^ which contains 74 555 transition metal complexes annotated with 20 quantum mechanical properties. We matched the ligand SMILES to our coordination number dataset and obtained a final dataset of 63 466 annotated transition metals. On this dataset, we tested the fingerprints using 1024-bit sizes for the ligand fingerprint, which was the best performing in the previous tasks. For all regression tasks we evaluated the *R*^2^ in a three-fold cross-validation.

Table 3 summarises the results for ELECTRUM alongside the two baseline encodings: Ligands and Atomic fingerprints. Across most properties, ELECTRUM outperforms both baselines, confirming the previous findings that the explicit metal electron configuration encoding provides additional predictive signal beyond ligand structure alone. The strongest performance (*R*^2^ > 0.80) is achieved for thermodynamic corrections and extensive additive properties such as enthalpy and Gibbs energy corrections, polarizability, and zero-point energy (ZPE) corrections. These quantities are largely determined by the overall atomic composition and bonding environment, features that are captured by the combined ligand–metal representation.

**Table 3:** *R* ^2^ value on the prediction of quantum chemical properties of the tmQMg dataset using the MLP model and ELECTRUM fingerprint (598 bits) compared to the ligands fingerprint without metal encoding (Ligands) as a negative control and the ligands fingerprint + a single scalar metal identifier (Atomic). Metrics for shorter and longer fingerprints can be found in the SI

Target	Ligands	Atomic	ELECTRUM
Dipole moment delta	0.325 ± 0.001	0.298 ± 0.016	**0.454** ± **0.009**
Dispersion energy delta	**0.678** ± **0.017**	−1.624 ± 1.657	0.288 ± 0.062
Electronic energy delta	0.762 ± 0.002	0.778 ± 0.002	**0.806** ± **0.002**
Enthalpy energy	0.300 ± 0.008	0.495 ± 0.009	**0.521** ± **0.026**
Enthalpy energy correction	0.844 ± 0.003	0.822 ± 0.005	**0.845** ± **0.006**
Entropy	0.794 ± 0.005	0.798 ± 0.012	**0.801** ± **0.004**
Gibbs energy	0.317 ± 0.011	0.510 ± 0.018	**0.519** ± **0.015**
Gibbs energy correction	0.843 ± 0.006	0.783 ± 0.049	**0.844** ± **0.002**
Heat capacity	0.820 ± 0.006	0.819 ± 0.003	**0.824** ± **0.006**
Highest vibrational frequency	0.281 ± 0.057	0.294 ± 0.052	**0.396** ± **0.023**
HOMO–LUMO gap delta	0.345 ± 0.003	0.154 ± 0.039	**0.582** ± **0.026**
Lowest vibrational frequency	0.408 ± 0.015	0.396 ± 0.003	**0.451** ± **0.012**
Polarisability	0.812 ± 0.004	0.808 ± 0.005	**0.827** ± **0.004**
TZVP dipole moment	0.398 ± 0.009	0.394 ± 0.009	**0.434** ± **0.015**
TZVP dispersion energy	**0.798** ± **0.007**	0.733 ± 0.051	0.782 ± 0.014
TZVP electronic energy	0.299 ± 0.019	0.487 ± 0.021	**0.520** ± **0.023**
TZVP HOMO energy	0.230 ± 0.015	0.121 ± 0.124	**0.288** ± **0.015**
TZVP HOMO–LUMO gap	0.515 ± 0.005	0.470 ± 0.065	**0.583** ± **0.020**
TZVP LUMO energy	0.320 ± 0.003	0.240 ± 0.058	**0.344** ± **0.024**
ZPE correction	0.846 ± 0.002	0.800 ± 0.034	**0.849** ± **0.004**

Moderate performance is observed for orbital energy gaps (*R*^2^ ≈ 0.58 for the HOMO–LUMO gap delta) while vibrational frequencies, dipole moments, and frontier orbital energies individually show lower *R*^2^ values (0.28–0.45), likely reflecting their dependence on 3D geometrical effects and higher-order electronic structure not explicitly represented in ELECTRUM. The moderate but non-zero predictive power in these cases suggests that some geometric and electronic effects are indirectly reflected in the ligand and metal features, but without the resolution achievable from 3D descriptors or quantum-derived inputs.

Overall, these results confirm that ELECTRUM provides a chemically meaningful representation capable of supporting both classification and regression tasks. The variation in performance across properties reflects the balance between chemical information captured by the fingerprint and the intrinsic complexity of the target. While state-of-the-art graph neural networks and 3D descriptor-based approaches often achieve higher accuracy for these properties (*R*^2^ ≈ 0.7–0.9 on comparable datasets), these methods require substantially more input information (*e.g.*, optimized 3D geometries) and computational effort. In contrast, ELECTRUM predictions are obtained from 2D ligand SMILES and a simple metal electron configuration encoding, with no geometry optimization or quantum chemical preprocessing. Moreover, we did not perform any hyperparameter tuning or model optimization for these tasks, as the aim here is not to compete directly with specialised prediction frameworks, but to demonstrate that ELECTRUM encodes chemically meaningful information that supports reasonable predictive performance across a range of properties.

While this test case demonstrates that ELECTRUM captures predictive signal for certain quantum mechanical properties, we think a more interesting validation involves its application to data-driven exploration of organometallic compounds with experimentally relevant properties, such as catalytic or biological activity. By generating chemically informative fingerprints for large datasets, ELECTRUM can support high-throughput virtual screening and the prioritization of candidates for synthesis and experimental evaluation. In this context, it is an important next step to assess the value of ELECTRUM through its ability to guide discovery workflows, where it is used to build models in low-data regimes and to identify promising compounds for experimental testing.

## Conclusion

With ELECTRUM, we introduce a fingerprint designed to overcome the challenges of representing transition metal complexes for machine learning applications. ELECTRUM can be generated using only the identity of the metal centre and the structures of the coordinating ligands. Despite this simplicity we show that ELECTRUM can be used to predict both coordination numbers and oxidation states of transition metal complexes from real datasets derived from the CSD.

While the datasets, evaluation protocols, and property definitions used in the literature vary substantially, making direct metric-to-metric comparison inappropriate, ELECTRUM's performance sits within the range of state-of-the-art approaches reported for related tasks. In oxidation state classification, Jablonka *et al.*^51^ report precision and recall above 95% using models that explicitly incorporate 3D geometric features of the local coordination environment, and Vela *et al.*'s^52^ cell2mol framework achieves comparable overall classification accuracy on crystallographic data. Earlier descriptor-based approaches such as the MCDL-25 representation have also been used successfully to predict quantum–mechanical properties of metal complexes with artificial neural networks, but they require either experimentally measured or computationally derived descriptors for each complex, which can be costly to obtain at scale. In our study, ELECTRUM achieves precision and recall above 91% for oxidation state prediction without 3D inputs or quantum-calculated descriptors, relying solely on 2D ligand SMILES and a binary encoding of the metal's electron configuration. Coordination number prediction likewise exceeds 85% ROC AUC and 74% F1 score, despite being evaluated on a much larger and more chemically diverse dataset than those typically used for geometry-aware descriptors. These comparisons suggest that ELECTRUM can approach the predictive power of more computationally intensive, geometry- or QM-dependent representations while requiring only minimal structural information.

Because ELECTRUM fingerprints are computed directly from ligand SMILES and a pre-tabulated electron configuration vector, their generation cost scales linearly with the number of ligands and does not require graph traversal of full 3D geometries or descriptor lookups from expensive calculations. This makes ELECTRUM particularly well-suited for large-scale “big data” applications, such as screening millions of hypothetical complexes or processing entire crystallographic databases, where the simplicity of the encoding translates directly into lower computational cost and shorter end-to-end ML pipelines.

Looking ahead, our goal is to further validate ELECTRUM across additional datasets. In particular, we aim to use ELECTRUM to train machine learning models aimed at predicting the biological properties of transition metal complexes.^3^ One key limitation of ELECTRUM in its current form is its inability to capture stereochemistry. For instance, the encoding for both cisplatin and transplatin would result in the exact same fingerprint in the current implementation. A promising avenue to address this, without substantially compromising computational efficiency, would be to incorporate chiral tags on selected ligand atoms involved in the metal coordination prior to hashing. This approach would allow stereoisomers to produce distinct fingerprints while preserving overall similarity and the rapid performance that enables ELECTRUM to scale to large datasets.

We hope to be able to expand ELECTRUM in the future to encode the complex stereochemistry of metal complexes. We surmise that the electron-configuration encoding of elements could also be a useful starting point for message passing graph-based approaches.

## Author contributions

A. F. conceived of the ELECTRUM approach. M. O. and A. F. generated the dataset. M. O. wrote the code. All authors contributed to the manuscript writing.

## Conflicts of interest

There are no conflicts to declare.

## Supplementary Material

DD-004-D5DD00145E-s001

## Data Availability

Data for this article, including the coordination number, oxidation state and tmQMg datasets are available at Frei Lab GitHub repository: https://github.com/TheFreiLab/electrum_val at https://doi.org/10.5281/zenodo.17205951. Supplementary information is available. See DOI: https://doi.org/10.1039/d5dd00145e.
