# Supplementary material for: ELECTRUM: an electron configuration-based universal metal fingerprint for transition metal compounds
Source: Digit Discov. 2025 Oct 20;4(12):3567–77. doi: 10.1039/d5dd00145e (PMC12548721; doi:10.1039/d5dd00145e)
Supplement: DD-004-D5DD00145E-s001 [file DD-004-D5DD00145E-s001.pdf]

Supplementary Information (SI) for Digital Discovery.
This journal is © The Royal Society of Chemistry 2025

Supporting information for: ELECTRUM: An Electron Configuration-based Universal Metal Fingerprint for Transition Metal Compounds

Markus Orsi[1], Angelo Frei[1,2*]*

[1] Department of Chemistry, Biochemistry & Pharmaceutical Sciences, University of Bern, Freiestrasse 3, 3012 Bern, Switzerland
[2] Department of Chemistry, University of York, York YO10 5DD, U.K.

E-mail:

angelo.frei@york.ac.uk

markus.orsi@unibe.ch

Table S1. Performance metrics for oxidation state classification using the MLP model and ELECTRUM fingerprint (598 bits) compared to the ligands fingerprint without metal encoding (Ligands) as a negative control and the ligands fingerprint + a single scalar metal identifier

Fingerprint	ROC AUC	PRC AUC	Precision	Recall	F1
**Ligands (256 bits)**	79.4 ± 0.31	47.9 ± 0.37	66.9 ± 0.82	64.3 ± 0.55	65.3 ± 0.36
**Atomic (256 bits)**	81.8 ± 0.38	52.8 ± 0.73	71.0 ± 1.26	68.6 ± 0.63	69.6 ± 0.59
**ELECTRUM (256 bits)**	95.6 ± 0.21	86.5 ± 0.35	**93.0 ± 0.30**	92.1 ± 0.41	92.5 ± 0.18
**Ligands (1024 bits)**	80.4 ± 0.33	49.5 ± 0.95	68.0 ± 1.39	66.0 ± 0.56	66.8 ± 0.83
**Atomic (1024 bits)**	82.1 ± 0.72	53.7 ± 0.97	72.2 ± 2.60	69.1 ± 1.32	70.3 ± 0.79
**ELECTRUM (1024 bits)**	95.5 ± 0.26	86.2 ± 0.58	92.8 ± 1.10	92.0 ± 0.48	92.4 ± 0.33

(Atomic).

Table S2. Performance metrics for coordination number classification using the MLP model and ELECTRUM fingerprint (598 bits) compared to the ligands fingerprint without metal encoding (Ligands) as a negative control and the ligands fingerprint + a single scalar metal identifier (Atomic).

Fingerprint	ROC AUC	PRC AUC	Precision	Recall	F1
**Ligands (256 bits)**	77.7 ± 0.56	40.5 ± 0.47	61.5 ± 0.18	58.6 ± 1.13	59.8 ± 0.65
**Atomic (256 bits)**	79.9 ± 0.19	44.9 ± 0.22	65.0 ± 0.23	62.7 ± 0.36	63.7 ± 0.14
**ELECTRUM (256 bits)**	**84.9 ± 0.13**	**56.2 ± 0.29**	73.1 ± 0.72	**71.9 ± 0.24**	**72.4 ± 0.21**
**Ligands (1024 bits)**	80.9 ± 0.27	47.0 ± 0.35	67.1 ± 0.49	64.6 ± 0.55	65.6 ± 0.31
**Atomic (1024 bits)**	82.1 ± 0.48	49.4 ± 0.55	68.8 ± 1.82	66.7 ± 0.97	67.5 ± 0.45
**ELECTRUM (1024 bits)**	**86.5 ± 0.23**	**60.6 ± 0.25**	77.0 ± 0.32	**74.8 ± 0.45**	**75.8 ± 0.14**

Table S3. Performance metrics for oxidation state classification with scrambled labels using the MLP model and ELECTRUM fingerprint.

Fingerprint	ROC AUC	PRC AUC	Precision	Recall	F1
**Ligands (256 bits)**	49.8 ± 0.21	14.2 ± 0.08	13.9 ± 0.29	13.9 ± 0.27	13.8 ± 0.33
**Atomic (256 bits)**	49.9 ± 0.09	14.3 ± 0.04	14.2 ± 0.22	14.2 ± 0.16	14.1 ± 0.20
**ELECTRUM (256 bits)**	50.2 ± 0.11	14.3 ± 0.03	14.6 ± 0.32	14.5 ± 0.22	14.5 ± 0.25
**Ligands (512 bits)**	49.9 ± 0.14	14.3 ± 0.02	14.1 ± 0.37	14.2 ± 0.28	14.0 ± 0.33
**Atomic (512 bits)**	49.9 ± 0.13	14.3 ± 0.05	14.1 ± 0.15	14.1 ± 0.17	14.0 ± 0.19
**ELECTRUM (512 bits)**	50.0 ± 0.24	14.3 ± 0.03	14.4 ± 0.68	14.4 ± 0.45	14.2 ± 0.52
**Ligands (1024 bits)**	50.1 ± 0.17	14.3 ± 0.05	14.5 ± 0.23	14.5 ± 0.27	14.4 ± 0.24
**Atomic (1024 bits)**	50.1 ± 0.21	14.3 ± 0.04	14.4 ± 0.49	14.4 ± 0.36	14.2 ± 0.29
**ELECTRUM (1024 bits)**	49.9 ± 0.12	14.3 ± 0.01	14.1 ± 0.32	14.1 ± 0.24	14.0 ± 0.40

Table S4. Performance metrics for coordination number classification with scrambled labels using the MLP model and ELECTRUM fingerprint.

Fingerprint	ROC AUC	PRC AUC	Precision	Recall	F1
**Ligands (256 bits)**	50.0 ± 0.07	9.1 ± 0.02	9.1 ± 0.14	9.1 ± 0.11	9.1 ± 0.12
**Atomic (256 bits)**	50.0 ± 0.03	9.1 ± 0.01	9.0 ± 0.07	9.0 ± 0.05	8.9 ± 0.08
**ELECTRUM (256 bits)**	50.0 ± 0.01	9.1 ± 0.01	9.1 ± 0.03	9.1 ± 0.01	9.1 ± 0.04
**Ligands (512 bits)**	50.0 ± 0.08	9.1 ± 0.02	9.1 ± 0.14	9.1 ± 0.13	9.0 ± 0.14
**Atomic (512 bits)**	50.0 ± 0.05	9.1 ± 0.01	9.1 ± 0.10	9.1 ± 0.08	9.1 ± 0.10
**ELECTRUM (512 bits)**	50.0 ± 0.03	9.1 ± 0.02	9.0 ± 0.03	9.0 ± 0.04	9.0 ± 0.03
**Ligands (1024 bits)**	50.0 ± 0.05	9.1 ± 0.01	9.1 ± 0.13	9.1 ± 0.09	9.0 ± 0.12
**Atomic (1024 bits)**	50.0 ± 0.07	9.1 ± 0.00	9.1 ± 0.17	9.1 ± 0.14	9.1 ± 0.15
**ELECTRUM (1024 bits)**	50.0 ± 0.04	9.1 ± 0.02	9.1 ± 0.07	9.1 ± 0.06	9.0 ± 0.06

Table S5. Performance metrics for oxidation state classification (true and scrambled labels) using the kNN classifier with Manhattan distance and ELECTRUM fingerprint.

Fingerprint	ROC AUC	PRC AUC	Precision	Recall	F1
**Ligands (256 bits)**	72.7 ± 0.29	39.2 ± 0.30	63.9 ± 0.21	52.7 ± 0.54	57.0 ± 0.34
**Atomic (256 bits)**	82.5 ± 0.70	58.3 ± 1.73	79.4 ± 1.12	69.6 ± 1.32	73.7 ± 1.26
**ELECTRUM (256 bits)**	**83.9 ± 0.50**	**60.8 ± 1.35**	**80.5 ± 0.94**	**72.0 ± 0.94**	**75.6 ± 0.98**
**Ligands (512 bits)**	72.5 ± 0.03	38.9 ± 0.32	63.7 ± 0.76	52.4 ± 0.05	56.6 ± 0.22
**Atomic (512 bits)**	82.3 ± 0.44	58.0 ± 1.08	79.3 ± 0.93	69.3 ± 0.82	73.5 ± 0.80
**ELECTRUM (512 bits)**	83.5 ± 0.54	60.2 ± 1.45	80.3 ± 0.99	71.4 ± 1.02	75.2 ± 1.05
**Ligands (1024 bits)**	72.5 ± 0.15	38.7 ± 0.63	63.3 ± 1.11	52.3 ± 0.27	56.5 ± 0.53
**Atomic (1024 bits)**	82.0 ± 0.61	57.4 ± 1.43	78.9 ± 1.00	68.8 ± 1.11	73.0 ± 1.08
**ELECTRUM (1024 bits)**	83.3 ± 0.58	59.7 ± 1.45	79.9 ± 0.92	71.0 ± 1.08	74.8 ± 1.06
**Ligands (256 bits) scr.**	50.0 ± 0.12	14.3 ± 0.05	14.0 ± 0.36	14.2 ± 0.16	12.8 ± 0.11
**Atomic (256 bits) scr.**	50.0 ± 0.05	14.3 ± 0.02	14.3 ± 0.31	14.3 ± 0.08	12.8 ± 0.08
**ELECTRUM (256 bits) scr.**	50.0 ± 0.08	14.3 ± 0.02	14.8 ± 0.46	14.3 ± 0.15	12.8 ± 0.18
**Ligands (512 bits) scr.**	50.0 ± 0.16	14.3 ± 0.06	14.4 ± 0.54	14.3 ± 0.22	12.8 ± 0.23
**Atomic (512 bits) scr.**	50.0 ± 0.05	14.3 ± 0.02	14.7 ± 0.66	14.4 ± 0.07	13.0 ± 0.10
**ELECTRUM (512 bits) scr.**	50.1 ± 0.08	14.3 ± 0.02	14.6 ± 0.24	14.5 ± 0.15	13.0 ± 0.20
**Ligands (1024 bits) scr.**	50.0 ± 0.07	14.3 ± 0.03	13.9 ± 0.34	14.3 ± 0.09	12.7 ± 0.08
**Atomic (1024 bits) scr.**	50.0 ± 0.09	14.3 ± 0.03	14.3 ± 0.25	14.3 ± 0.13	12.8 ± 0.13
**ELECTRUM (1024 bits) scr.**	49.9 ± 0.14	14.3 ± 0.05	14.1 ± 0.60	14.2 ± 0.19	12.7 ± 0.28

Figure S1. Composition of the coordination number dataset. a) Number of complexes per metal, sorted by frequency. b) Distribution of coordination numbers across all complexes. c) Cross-tabulation heatmap showing the number of complexes for each metal–coordination number combination.

Figure S2. Composition of the oxidation state dataset. a) Number of complexes per metal, sorted by frequency. b) Distribution of oxidation states across all complexes. c) Cross-tabulation heatmap showing the number of complexes for each metal–oxidation state combination.
